# Supplementary figures and images for: How much does it cost to save a species from extinction? Costs and rewards of conserving the Lear's macaw
Source: R Soc Open Sci. 2019 Jul 10;6(7):190190. doi: 10.1098/rsos.190190 (PMC6689625; doi:10.1098/rsos.190190)

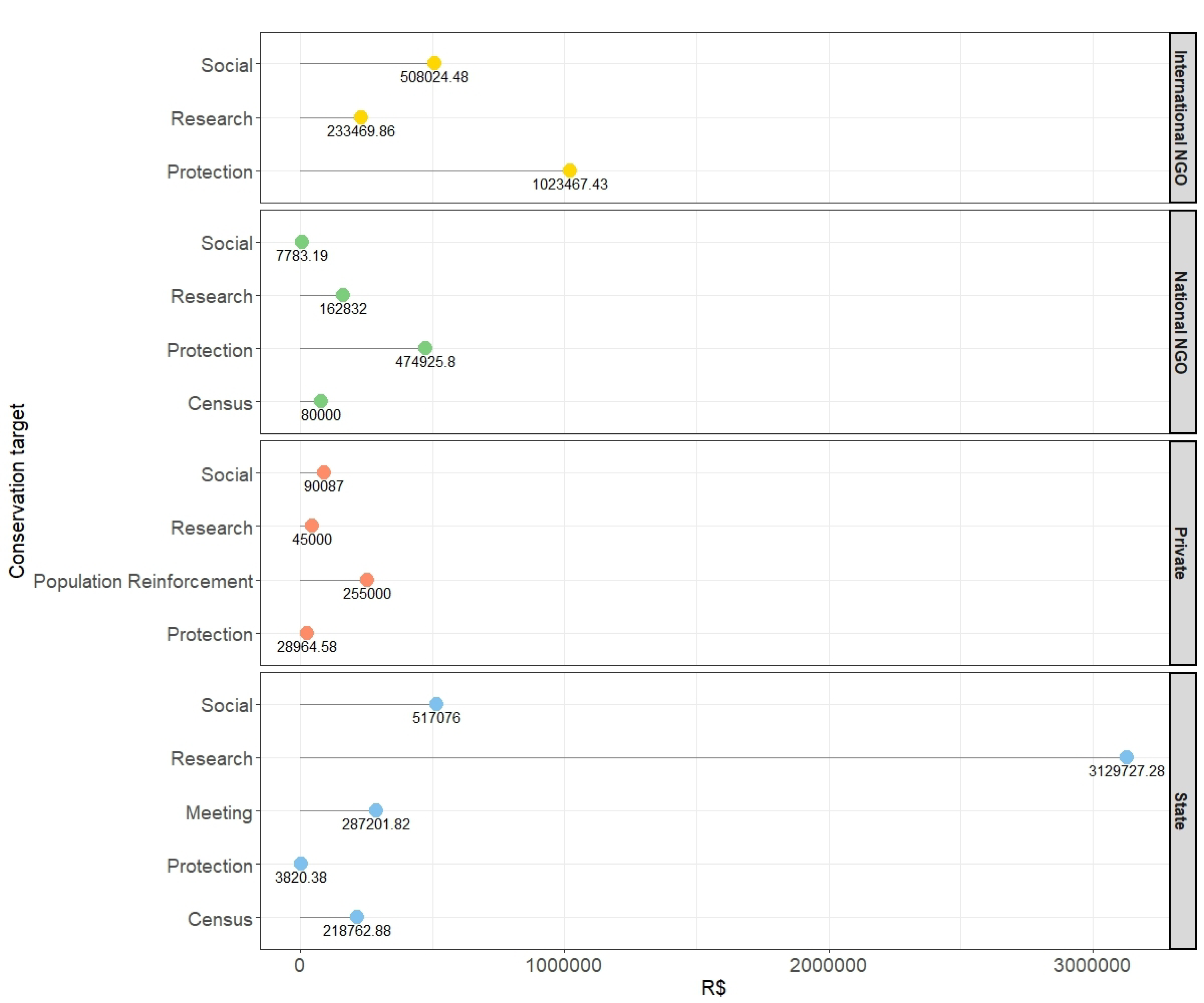

Supplement: Figure S1 [file rsos190190supp1.tif]
